# Characterization of Biological Properties of Individual Phenolamides and Phenolamide-Enriched Leaf Tomato Extracts

**DOI:** 10.3390/molecules28041552

**Published:** 2023-02-06

**Authors:** Marwa Roumani, Armelle Ropars, Christophe Robin, Raphaël E. Duval, Jean-Pol Frippiat, Michel Boisbrun, Romain Larbat

**Affiliations:** 1 Université de Lorraine, INRAE, LAE, F-54000 Nancy, France; 2 Université de Lorraine, SIMPA, F-54000 Nancy, France; 3 Université de Lorraine, CNRS, L2CM, F-54000 Nancy, France; 4 Institut Agro, University of Angers, INRAE, IRHS, SFR QUASAV, F-49000 Angers, France

**Keywords:** tomato, *Tuta absoluta*, phenolamide, cytotoxicity, anti-inflammatory, biovalorization

## Abstract

Resistance to conventional treatments renders urgent the discovery of new therapeutic molecules. Plant specialized metabolites such as phenolamides, a subclass of phenolic compounds, whose accumulation in tomato plants is mediated by the biotic and abiotic environment, constitute a source of natural molecules endowed with potential antioxidant, antimicrobial as well as anti-inflammatory properties. The aim of our study was to investigate whether three major phenolamides found in *Tuta absoluta*-infested tomato leaves exhibit antimicrobial, cytotoxic and/or anti-inflammatory properties. One of them, *N*^1^,*N*^5^,*N*^14^-tris(dihydrocaffeoyl)spermine, was specifically synthesized for this study. The three phenolamides showed low to moderate antibacterial activities but were able to counteract the LPS pro-inflammatory effect on THP-1 cells differentiated into macrophages. Extracts made from healthy but not *T. absoluta*-infested tomato leaf extracts were also able to reduce inflammation using the same cellular approach. Taken together, these results show that phenolamides from tomato leaves could be interesting alternatives to conventional drugs.

## 1. Introduction

Globally, the human population is facing an increasing number of public health problems. Cancer is surely among the critical threats due to its high frequency and because the number of new cases increases each year [1,2,3]. Another serious problem concerns infectious diseases and notably zoonotic diseases that increase with the rise of population density and climate change [4,5]. In addition, population ageing and/or lack of exercise, obesity, unhealthy food, and environmental factors such as pollution promote a rise of chronic inflammatory diseases [6,7,8].

For most of these diseases, therapeutic resistance constitutes a significant challenge. One of the major problems of this therapeutic resistance is encountered with glucocorticoids. Indeed, patients suffering from lymphoid malignancies or chronic diseases such as inflammatory bowel disease (IBD) and asthma are classically treated with glucocorticoids, but a significant portion of patients become resistant to the treatment [9,10,11]. Unfortunately, therapeutic resistance in cancers is not limited to glucocorticoids because chemotherapeutic agents or novel targeted drugs are affected by the mechanism of multidrug resistance (MDR). Approximately 90% of deaths are due to this acquired resistance [12,13]. Cancers and chronic inflammation diseases are not the only ones for which therapeutic resistance emerges. Indeed, antifungal, antiviral and especially antibacterial resistance also poses a major challenge. This antibiotic resistance is in large part due to the misuse of these molecules and global warming also increases this resistance [14,15,16]. In light of all these data, it appears urgent and crucial to discover new classes of bioactive molecules able to challenge conventional drugs. In this research area, plants open a vast field of discovery due to their high variety and the huge diversity of molecules that they can produce. Plant specialized metabolites represent a set of molecules of various structures and functions, necessary for the plant to interact with its environment [17]. They present diverse biological properties and constitute an important potential of active principles, notably in the health sector. Currently, it is estimated that more than 26,000 plant species are used in medicine [18]. This number has increased in recent decades and is expected to further increase due to the progress made in the identification of new functionalities and the above-mentioned challenges faced by health systems.

Biosynthesis of specialized metabolites in plants is mediated by pedoclimatic conditions and biotic interactions [19]. Thus, studying plant metabolome under different environmental conditions is likely to reveal a greater diversity of specialized metabolites and associated therapeutic properties. The phenolic compounds family seems the most promising types of metabolites, with an extensive literature describing their antioxidant, anti-inflammatory, neuroprotective and anticancer properties [20,21,22,23,24]. In addition, dietary phenolics are essential to maintain a healthy gut microbiome [25].

**Figure 1:**
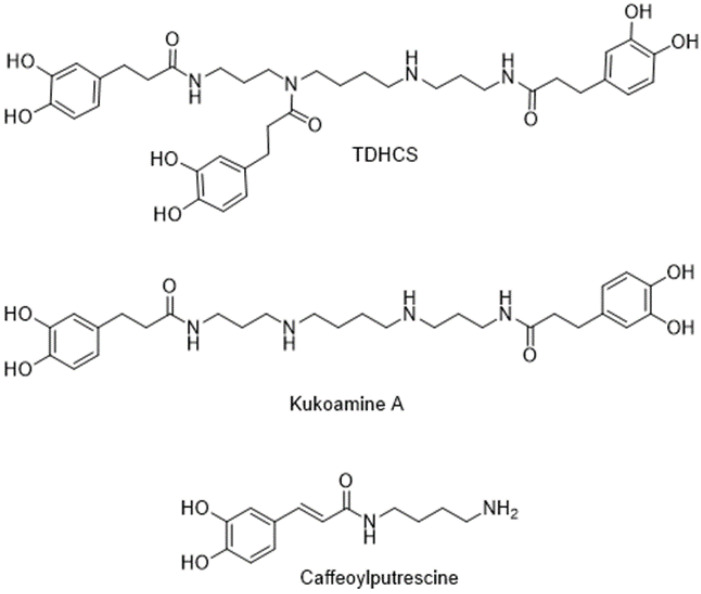
Chemical structure of the main phenolamides studied in this work.

In this context, we have recently characterized the metabolic response of tomato plants (*Solanum lycopersicum* L.) to the herbivory of one of its major pests, the lepidoptera *Tuta absoluta*, also known as tomato leaf miner [26]. Combining untargeted metabolomics and transcriptomics analyses, we revealed that the herbivory of a dozen of *T. absoluta* larvae restricted to a single leaf led to the systemic accumulation of phenolics including a large diversity of phenolamides [26]. Phenolamides, also called hydroxycinnamic acid amides, are found in floral organs and pollen of Angiosperms, and also in vegetative organs of Brassicaceae, Solanaceae and Poaceae, where they are associated with plant resistance to biotic and abiotic stresses [27,28]. They are composed of the association of hydroxycinnamic acids (*p*-coumaric, caffeic, and ferulic acids) with a mono/polyamine (putrescine, agmatine, spermine, spermidine, noradrenaline, octopamine, etc.), giving rise to a large diversity of structures [29]. In relation to their structural diversity, phenolamides exhibit a large panel of biological activities including anticancer, anti-inflammatory, antioxidant and neuroprotective effects highlighted in two recent reviews [30,31]. Phenolamides composed of putrescine and spermine as amine moieties were reported to have a large range of these activities [31]. Putrescine and spermine derivatives are found in infested tomato plants. In fact, among the 45 phenolamides whose accumulation increased in response to *T. absoluta* infestation in tomato, 14 were composed of putrescine and 5 of spermine [26]. Among the latter group of spermine derivatives, we were able to identify the rare phenolamide *N*^1^*,N*^5^*,N*^14^-tris(dihydrocaffeoyl)spermine (TDHCS, Figure 1) as the most accumulated phenolamide in infested tomato tissues. To our knowledge, no data exist on its potential biological activities. Interestingly, TDHCS is structurally close to kukoamine A (Kuk_A, also found in infested tomato tissues, Figure 1), which is under study as a neuroprotective treatment for Parkinson’s disease and is an inhibitor of human glioblastoma cell growth and migration in an in vitro model [32,33]. Caffeoylputrescine (CP, Figure 1) also constituted a major phenolamide accumulation in infested tomato leaves for which only limited data are available regarding its biological properties [34,35,36].

This study investigated the antimicrobial, cytotoxic and anti-inflammatory properties of Kuk_A, CP and TDHCS which accumulate in infested tomato leaves. As TDHCS is not commercially available, we chemically synthesized it from spermine thanks to an innovative chemical strategy. In addition, we investigated if tomato leaf extracts provided from healthy or infested plants presented anti-inflammatory activities.

## 2. Results

### 2.1. Phenolic Composition of Tomato Leaves under T. absoluta Herbivory

**Figure 2:**
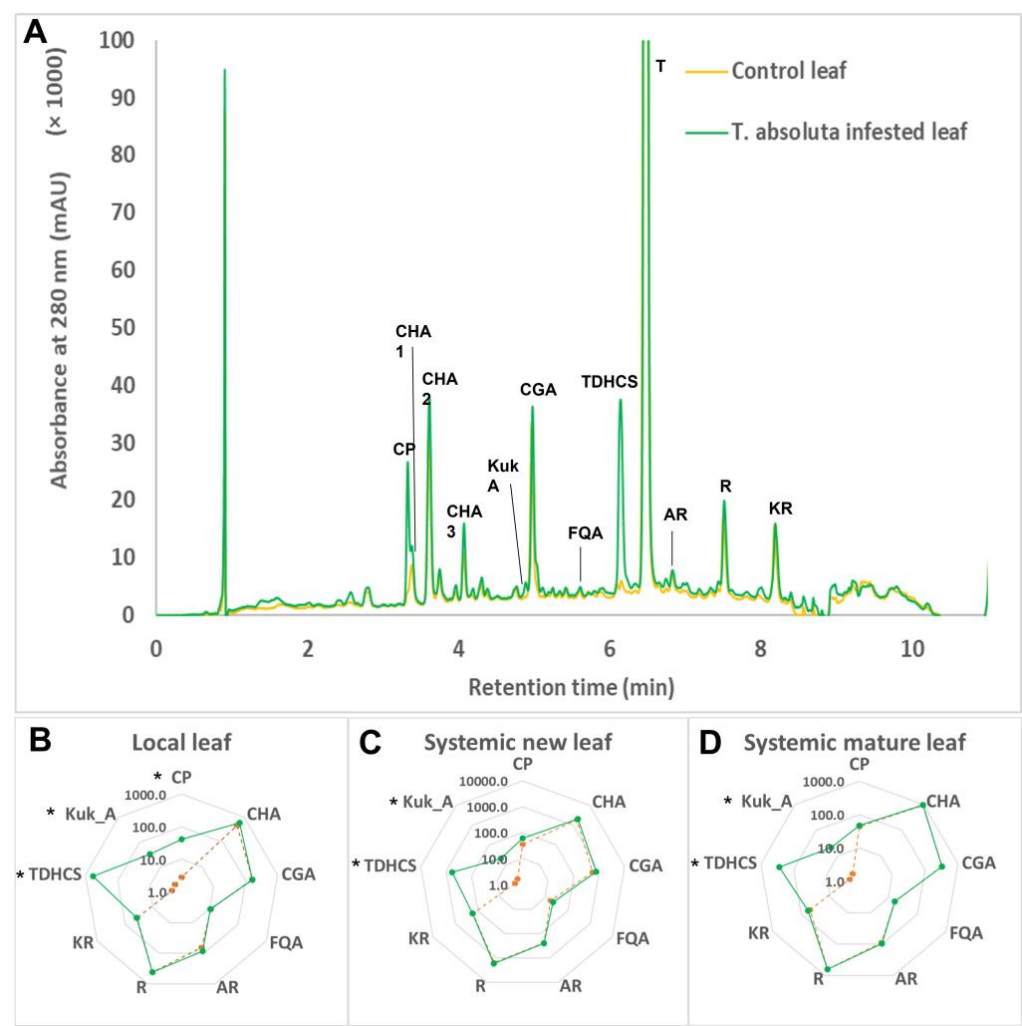
Impact of *T. absoluta* herbivory on the phenolic composition of tomato leaf. (**A**) Chromatogram at 280 nm of tomato local leaf extracts with and without infestation with *T. absoluta*. The phenolic compounds have been identified according to Larbat et al., 2014 [37]. CP, caffeoylputrescine; CHA1-3, caffeoylhexaric acid isomers 1 to 3; CGA, chlorogenic acid; FQA, feruloylquinic acid; TDHCS, *N*^1^*,N*^5^*,N*^14^-tris*(*dihydrocaffeoyl*)*spermine; Kuk_A, kukoamine A; AR, apio-furanosyl rutine; R, rutine; KR, kaempferol rutinoside. Taxifoline (T) was used as an internal standard (**B**–**D**). Concentrations (µg/g dry weight) of the phenolic compounds identified in leaves infested with *T. absoluta* larvae (**B**, local leaf), in leaves above the leaf range infested with *T. absoluta* larvae (**C**, systemic new leaf) and in leaves below the leaves infested with *T. absoluta* (**D**, systemic mature leaf). Values are the means of six replicates. Asterisks (*) indicate metabolites with concentrations significantly different between healthy and *T. absoluta*-infested tomato (*p* < 0.05).

Eleven phenolic compounds were detected and quantified from tomato leaves. They fall into three subclasses, namely the hydroxycinnamic acid derivatives, which include three caffeoyl hexaric acids isomers (CHA1-3), chlorogenic acid (CGA) and feruloyl quinic acid (FQA); three flavonoids identified as rutin (R), kampferol rutinoside (KR) and apio-furanosyl rutin (AR); and phenolamides with CP, Kuk_A and TDHCS (Figure 2A). Among all these compounds, *T. absoluta* infestation dramatically and specifically increased the phenolamide content in infested leaves (Figure 2B) but also in “systemic” leaves (leaves from infested plants that were not eaten by larvae) (Figure 2C,D). The impact of *T. absoluta* herbivory was more pronounced in infested leaves, since the 3 phenolamides followed were significantly induced. Among them, TDHCS was the most induced, with concentrations reaching 580 ± 121 µg/g DW in the local leaf of the infested plants, whereas it was almost not detectable (<1 µg/g DW) in healthy plants. In the same organs, Kuk_A and CP increased from 2 µg/g DW to approximately 35 µg/g DW (Figure 2B–D). In the systemic mature and new leaves, the increase in phenolamide was restricted to TDHCS and Kuk_A. By contrast, none of the hydroxycinnamoyl esters, nor those of the flavonoid subclass, were affected by *T. absoluta* herbivory (Figure 2). In this study, we focused on three phenolamides, CP, Kuk_A and TDHCS. Two of them, CP and Kuk_A, were commercially available, but TDHCS was not and was synthesized from the spermine motif.

### 2.2. Innovative Synthesis of N^1^,N^5^,N^14^-tris(dihydrocaffeoyl)spermine (TDHCS)

**Scheme 1:**
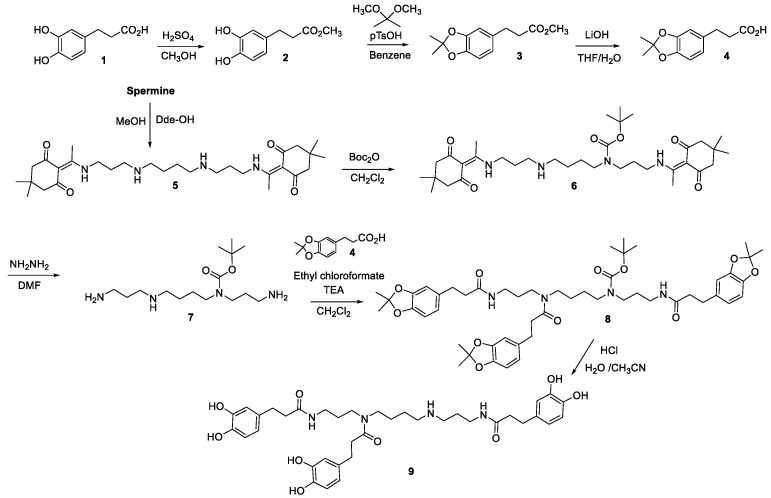
Synthetic strategy aiming to obtain *N*^1^*,N*^5^*,N*^14^-tris(dihydrocaffeoyl)spermine (TDHCS).

In a first attempt, we intended to prepare the titled compound via solid-supported organic synthesis, as reported by Yingyongnarongkul and colleagues [38]. In this study, careful application of this procedure afforded the desired compound together with the spermine scaffold exhibiting two, four or five dihydrocaffeoyl moieties instead of three. We thus moved towards a classical liquid organic synthesis. As Yingyongnarongkul’s team did, we first protected both ends of spermine with a 1-(4,4-dimethyl-2,6-dioxocyclohexylidene)ethyl (Dde) group [38], but we also protected the catechol part of the dihydrocaffeoyl moiety as an acetonide to avoid cross-reactions. The whole synthesis is depicted in Scheme 1. After esterification of dihydrocaffeic acid **1** [39] giving **2**, its catechol moiety was protected as an acetonide [40] to give **3**. The ester was subsequently saponified, affording the desired compound **4** in good overall yield. Both primary amino ends of spermine were easily protected with a Dde group giving compound **5**. Selective Boc protection of only one secondary amino group could be achieved by using 0.8 eq. of Boc_2_O, giving compound **6** in 74% yield. Dde deprotection gave compound **7,** which was subsequently reacted with **4** to afford the desired compound **8** in 20% yield over two steps. Acidic treatment resulted in the removal of both the Boc and the three acetonide protecting groups to give the final compound **9** in 37% yield after reversed-phase HPLC purification (Scheme 1).

### 2.3. Antibacterial Activity of the Three Phenolamides

The three pure compounds (Kuk_A, CP and TDHCS) inhibited the growth of several bacterial strains, but the minimum inhibitory concentrations (MICs) measured remained high. Kuk_A was the molecule with the broadest antibacterial spectrum, with MICs between 256 and 1024 mg/mL, but without any activity against *Klebsiella pneumonia*e (CMI > 1024 mg/mL). Conversely, CP was only active against Gram-positive bacteria (i.e., *S. aureus* and *E. faecalis*, with MICs = 512 and 1024 mg/mL, respectively), while TDHCS only inhibited the growth of *Acinetobacter baumannii* (MIC = 256 mg/L) (Supplementary Material Figure S1).

### 2.4. Anticancer Activity of TDHCS on MCF-7 and Caco-2 Cells

Anticancer activity of TDHCS (i.e., cell growth inhibition) was assessed using MTT assay. TDHCS exhibited weak anticancer activity on both MCF-7 and Caco-2 cells, with greater antiproliferative activity at higher concentrations (IC50 was only reached for concentrations above 100 µg/mL). This activity appeared to be dose dependent with cell viability falling to approximately 40% (Caco-2 cells) and 30% (MCF-7 cells) at a TDHCS concentration of 200 µg/mL (Supplementary Materials Figures S2 and S3).

### 2.5. Cytotoxicity and Anti-Inflammatory Activity of Three Phenolamides

THP-1 cells were differentiated into macrophages and used to analyze the potential anti-inflammatory effects of the three phenolamides (Kuk_A, CP and TDHCS) on LPS-induced pro-inflammatory response. Indeed, LPS is classically used to promote a pro-inflammatory response in in vitro studies, which is notably characterized by a strong increase in TNF-α production by cells [41,42]. Two conditions were used to study two different cellular states. The first condition consisted of pre-incubating differentiated THP-1 cells with 100 ng/mL of LPS to induce a pro-inflammatory state and, one hour later, to add one phenolamide at different concentrations (LPS + phenolamide). The purpose of this approach was to determine if the phenolamide was able to counteract LPS-induced pro-inflammatory response. The second condition was the opposite, consisting in incubating THP-1 cells with one phenolamide, to potentially promote an anti-inflammatory cellular state, and one hour later to add 100 ng/mL of LPS (phenolamide + LPS). As phenolamides could have deleterious effects on cell viability and thus, indirectly decrease TNF-α production, we first had to evaluate the viability of THP-1 cells incubated with different phenolamides concentrations in the presence of LPS or not. In both cases, cell viability and TNF-α production were analyzed after 24 h of treatment.

#### 2.5.1. Cytotoxicity of the Three Phenolamides on Differentiated THP-1 Cells

**Figure 3:**
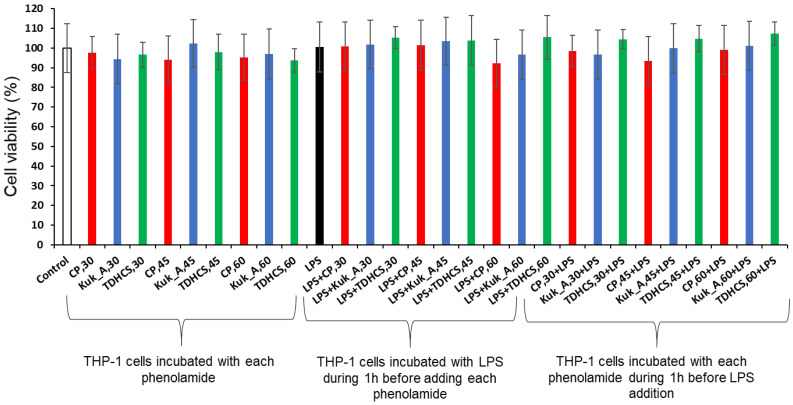
Effects of the three phenolamides on the viability of differentiated THP-1 cells. Four concentrations (30, 45 and 60 µM) of CP, Kuk_A and TDHCS were tested. Differentiated THP-1 cells were incubated either with these molecules alone or with 100 ng/mL of LPS added one hour before or after the phenolamide addition. Twenty-four hours later, THP-1 cell viability was analyzed by crystal violet assay and optical density (O.D.) was measured at 595 nm. This figure is representative of six independent experiments performed in triplicates. “Control” corresponds to a THP-1 cell culture in which no product was added (negative control). “LPS” corresponds to a THP-1 cell culture in which only LPS was added (positive control). “LPS + phenolamide, 30” indicates that LPS was added before phenolamide at a concentration of 30 µM while “phenolamide, 30 + LPS’ indicated that the phenolamide at a concentration of 30 µM was added before LPS. Data are presented as the mean ± S.D. *t*-tests were used to identify statistically significant differences.

Whatever the treatment and the concentrations tested, no alteration of THP-1 cell viability was detected using the crystal violet assay (Figure 3). Thus, the three phenolamides used at 30, 45 and 60 µM in the presence or absence of LPS had no impact on THP-1 cell viability (cell viability > 97%) and could be used for further experiments on THP-1 cells.

#### 2.5.2. Anti-Inflammatory Activity of the Three Phenolamides on Differentiated THP-1 Cells

**Figure 4:**
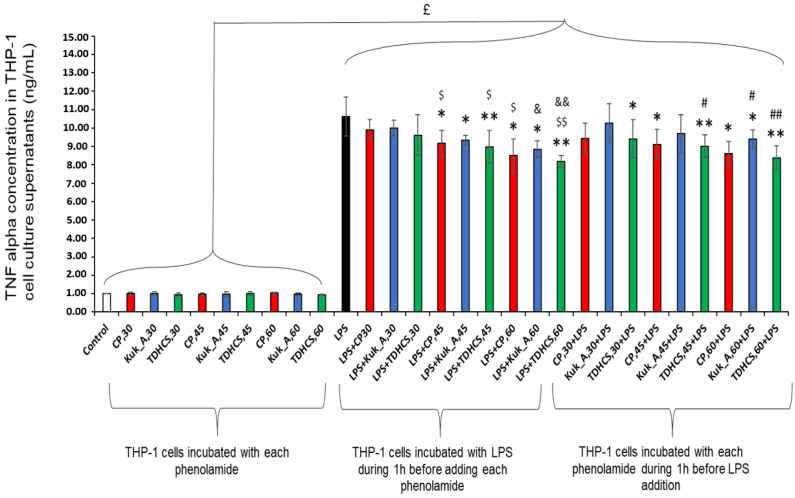
CP, Kuk_A and TDHCS are able to reduce the production of TNF-α induced by LPS in differentiated THP-1 cells. The three phenolamides were used at 30, 45 and 60 µM. Differentiated THP-1 cells were incubated for 24 h either with these molecules alone or with 100 ng/mL of LPS added one hour before or after the phenolamide addition. Cell culture supernatants were then collected and used to quantify TNF-α production by ELISA. This figure is representative of six independent experiments performed in triplicates. “Control” corresponds to a THP-1 cell culture in which no product was added (negative control). “LPS” corresponds to a THP-1 cell culture in which only LPS was added (positive control). “LPS + phenolamide, 30” indicates that LPS was added before phenolamide at a concentration of 30 µM while “phenolamide, 30 + LPS’ indicated that phenolamide at 30 µM was added before LPS. Data are presented as the mean ± S.D. *t*-tests were used to identify statistically significant differences. £ *p* < 0.05 vs. conditions without LPS; * *p* < 0.05, ** *p* < 0.01 vs. LPS; $ *p* < 0.05, $$ *p* < 0.01 vs. LPS + phenolamide, 30; & *p* < 0.05, && *p* < 0.01 vs. LPS + phenolamide, 45; # *p* < 0.05, ## *p* < 0.01 vs. phenolamine, 30 + LPS.

TNF-α secretion by THP-1 cells increased strongly when LPS was added to cell culture (indicated by “£” in Figure 4). Differentiated THP-1 cells produced less TNF-α when phenolamide was added at 45 or 60 µM one hour after or before LPS. This inhibitory effect was already observed for TDHCS at 30 µM when added before LPS (12% decrease in TNF-α production). THP-1 cells always produced less TNF-α in the presence of CP or TDHCS than in the presence of Kuk_A, whatever the concentration and the treatment (LPS added before or after phenolamide). When LPS was added before the phenolamide at 60 µM, TNF-α production was reduced by 17% with Kuk_A, whereas it was reduced by 21 and 23% with CP and TDHCS, respectively. When the phenolamide at 60 µM was added before LPS, TNF-α production was 12% lower with Kuk_A, whereas it was 20 and 21% lower with CP and TDHCS, respectively. This last observation was dependent of the phenolamide concentration; TNF-α production by THP-1 cells was reduced by 12, 16 and 21% with TDHCS concentrations of 30, 45 and 60 µM, respectively, when TDHCS was added before LPS. Pure phenolamides tested at 15 µM before or after LPS addition were unable to counteract LPS pro-inflammatory effect on THP-1 cells (data not shown). 

### 2.6. Cytotoxicity and Anti-Inflammatory Activity of Tomato Leaf Extracts on Differentiated THP-1 Cells

As Kuk_A, CP and TDHCS are present in tomato leaves and highly accumulate in leaves from tomatoes infested by *T. absoluta*, we investigated whether tomato leaf extracts from both healthy and *T. absoluta* infested plants have also anti-inflammatory activities on THP-1 cells. As for pure phenolamides, we first determined the impact of these extracts, at different concentrations, on THP-1 cell viability. Then, we investigated the impact of these extracts on the production of TNF-α by THP-1 cells. Chlorogenic acid (CGA) was used as reference in these experiments because it represents the major polyphenol (approximately 30%) of these tomato leaf extracts.

#### 2.6.1. Cytotoxicity of Tomato Leaf Extracts on Differentiated THP-1 Cells

**Figure 5:**
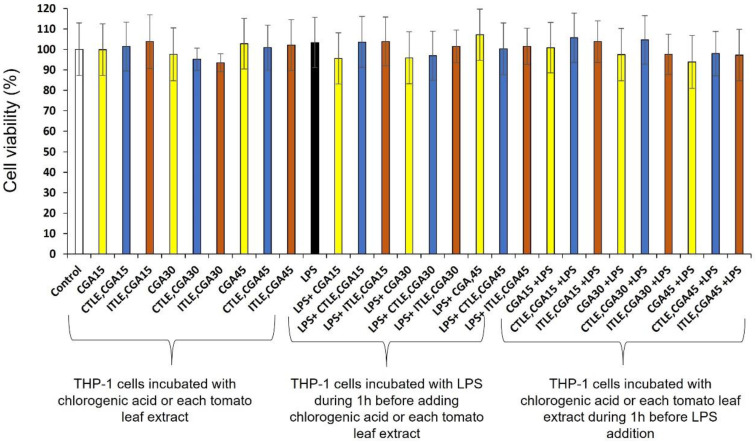
Effects of tomato leaf extracts on the viability of differentiated THP-1 cells. Extracts of tomato leaves infested (ITLE) or not (CTLE) with *T. absoluta* were evaluated. Three concentrations of tomato leaf extracts (50, 100 and 150 µg/mL) in which chlorogenic acid (CGA) concentrations, the major phenolic compound, represent, respectively, 15, 30 and 45 µg/mL were used. Differentiated THP-1 cells were incubated with CGA or a tomato leaf extract alone or with 100 ng/mL of LPS added one hour before or after the addition of the tomato leaf extract or CGA. Twenty-four hours later, THP-1 cell viability was analyzed by crystal violet assay and optical density (O.D.) was measured at 595 nm. “Control” corresponds to a THP-1 cell culture in which no product was added (negative control). “LPS” corresponds to a THP-1 cell culture in which only LPS was added (positive control). “LPS + tomato leaf extract,15” indicates that LPS was added before tomato leaf extract in which the CGA concentration was 15 µg/mL while “tomato leaf extract,15 + LPS” indicates that tomato leaf extract in which the CGA concentration was 15 µg/mL was added before LPS. This figure is representative of six independent experiments performed in triplicates. Data are presented as the mean ± S.D. *t*-tests were used to identify statistically significant differences.

Whatever the concentration and the treatment, both tomato leaf extracts had no impact on THP-1 cell viability (cell viability > 97%; Figure 5).

#### 2.6.2. Anti-Inflammatory Activity of Tomato Leaf Extracts on Differentiated THP-1 Cells

**Figure 6:**
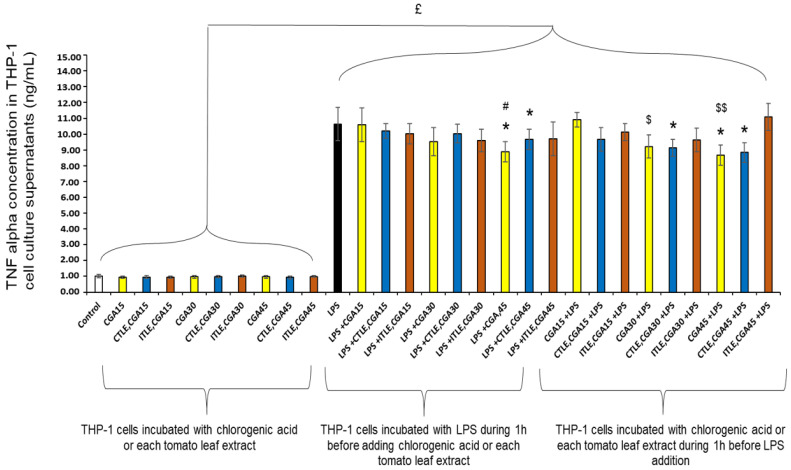
Anti-inflammatory activity of tomato leaf extracts and CGA on differentiated THP-1 cells. Extracts of tomato leaves infested (ITLE) or not (CTLE) with *T. absoluta* were evaluated. Three concentrations, 50, 100, and 150 µg/mL, respectively, corresponding to 15, 30 and 45 µg/mL of chlorogenic acid (CGA) were used. CGA was also used as a reference. Differentiated THP-1 cells were incubated for 24 h either with a tomato leaf extract, CGA alone or with 100 ng/mL of LPS added one hour before or after the addition of a tomato leaf extract or CGA. Cell culture supernatants were then collected and used to quantify TNF-α production by ELISA. “Control” corresponds to a THP-1 cell culture in which no product was added (negative control). “LPS” corresponds to a THP-1 cell culture in which only LPS was added (positive control). “LPS + tomato leaf extract,15” indicates that LPS was added before the tomato leaf extract in which the concentration of CGA is 15 µg/mL while “tomato leaf extract,15 + LPS” indicates that the tomato leaf extract containing CGA at 15 µg/mL was added before LPS. This figure is representative of six independent experiments performed in triplicates. Data are presented as the mean ± S.D. *t*-tests were used to identify statistically significant differences. £ *p* < 0.05 vs. conditions without LPS. * *p* < 0.05 vs. LPS alone; $ *p* < 0.05, $$ *p* < 0.01 vs. CGA,15 + LPS; # *p* < LPS + CGA, 30.

CGA, at a concentration of 45 µg/mL, was able to reduce TNF-α production when added after or before LPS (decreases of 17 and 19%, respectively; Figure 6). The control tomato leaf extract (CTLE) added one hour before or after LPS in which the CGA concentration was 45 µg/mL was also able to reduce TNF-α production (significant decreases of 10% and 17%, respectively). Moreover, CGA at 30 µg/mL and CTLE in which CGA was at 30 µg/mL added one hour before LPS were able to induce an anti-inflammatory state in THP-1 cells (TNF-α production decreased by 14% and 15%, respectively). These observations are likely due to chlorogenic acid already present in control tomato leaf extract (CTLE). However, the infested tomato leaf extract (ITLE) was unable to decrease LPS-induced TNF-α production when added before or after LPS, and whatever the concentration used.

## 3. Discussion

In the present study, we analyzed the biological properties of three major phenolamides, kukoamine A (Kuk_A), caffeoylputrescine (CP) and *N*^1^*,N*^5^*,N*^14^-tris(dihydrocaffeoyl)spermine (TDHCS), accumulated in leaves of tomato plants facing herbivory by the pest *T. absoluta*. First, as the phenolamide TDHCS was not commercially available, we synthesized it from the spermine motif. To our knowledge, it is the second time that its synthesis is published. Our protocol has the advantages of having a good yield and being relatively not expensive. We previously tried to reproduce the solid-phase method described in [38] but in this study, according to LC–MS analysis, it gave a mixture of spermine derivatives holding two to five dihydrocaffeoyl moieties instead of three. Indeed, on the one hand, this procedure involved the grafting of the di-protected (Dde) compound **5**, harboring two free secondary amines which may to some extent both graft on the resin. Such a double grafting will at the end of the synthesis result in only two dihydrocaffeoyl functionalization. On the other hand, this protocol did not involve any protection of the nucleophile catechol moieties. As a result, in addition to the secondary amines, they could also couple with the excess of activated dihydrocaffeic acid, yielding a final compound holding more than the expected three functionalizations. Consequently, we developed a new liquid-phase approach as follows. After the protection of both primary amino groups of spermine with Dde giving **5**, the coupling with a slight default of Boc_2_O gave **6**, holding only one free secondary amino group. Easy deprotection of both Dde-protected amino groups gave Boc-monoprotected **7**, exhibiting two primary and one secondary free amines, enabling their simultaneous coupling with a dihydrocaffeoyl moiety. In order to avoid any side-reaction with the catechol group, we easily prepared the acetonide-protected derivative **4**, giving, after coupling with **7**, the Boc- and acetonide-protected compound **8**. Final deprotection gave the expected pure *N*^1^*,N*^5^*,N*^14^-tris(dihydrocaffeoyl)spermine (TDHCS) **9**.

Then, we evaluated the anticancer activity as well as the potential cytotoxicity of this molecule on Caco-2 and MCF-7 cells. We showed that at concentrations below 100 µg/mL, TDHCS had no cancer activity, and did not induce any cytotoxicity. We further evaluated the potential antimicrobial activity of the three phenolamides against both Gram-negative and Gram-positive bacteria. Any of the three phenolamides exerted a strong antimicrobial activity against tested bacteria. Nevertheless, results obtained with *S. aureus* and *A. baumannii* are encouraging. Indeed, against *A. baumannii*, Kuk_A and TDHCS exerted a moderate antimicrobial activity, respectively, at 512 and 256 mg/L. Against *S. aureus*, CP and mainly Kuk_A exerted an antimicrobial activity with respective MIC at 512 and 256 mg/L. This slight antimicrobial activity observed with Kuk_A was also observed by Kyselka et al. (2018) [43]. However, to our knowledge, for CP and TDHCS, no data are available concerning their potential antimicrobial activity. *S. aureus* is a well-known bacterial pathogen responsible for skin and soft tissue infections that belongs to the ESKAPE group and has been classified as “high priority” due to its resistance to many antibiotics, such as methicillin [44,45] or more worryingly to vancomycin. Similarly, *A. baumannii* is an opportunistic pathogen bacterium that causes respiratory infections, wounds, or suppurative infections (e.g., abscesses) in any organ, including the lungs, urinary tract, skin and soft tissues. This bacterium has been classified as “critical priority” due to its very high resistance to carbapenems. Consequently, it would be interesting to modify the structures of these two phenolic compounds in order to increase their antimicrobial property. For example, through docking and binding sites analysis, it may be possible to determine which groups and/or amino-acids are essential for the biological properties of these biomolecules and then, modify them.

Given that many phenolic compounds present anti-inflammatory properties, we investigated whether the three phenolamides were able to counteract the pro-inflammatory action of LPS on THP-1 cells differentiated into macrophages, and compared their efficiency. Our data showed that when the three phenolamides were added one hour after LPS, they were able to diminish TNF-α production by differentiated THP-1 cells. Moreover, when added one hour before LPS, the three phenolamides were able to set differentiated THP-1 cells in an anti-inflammatory state that afterwards render them less sensible to the LPS pro-inflammatory stimulus. The ability of Kuk_A to counteract LPS-induced proinflammation was very recently described by Liu et al. and Wang et al. in other cells than macrophages [46,47]. Liu et al. used an in vivo mouse model of lung injury with LPS, and an in vitro model of alveolar epithelial cells preincubated with LPS. In both models, they demonstrated that Kuk_A decreased the expression of inflammatory factors and oxidative stress markers that were dependent of CCR5 inhibition [46]. In the same manner, Wang et al. demonstrated that Kuk_A added in cell culture medium of nucleus pulposus cells preincubated with LPS, attenuated LPS-induced apoptosis, extracellular matrix degradation, and inflammation. This inhibition was dependent of the activation of the PI3K/Akt pathway [47]. Thus, the ability of Kuk_A to diminish inflammation seems to be shared by different cell types, which is very promising in terms of future biomedical application. Our assays with CP and TDHCS highlighted that these two phenolamides were endowed with more potent anti-inflammatory properties than Kuk_A. To our knowledge, it is the first time that this observation is made for these two phenolamides.

The fact that the three phenolamides are able to inhibit LPS-induced inflammation in THP-1 macrophages is important because macrophages constitute the first line of defense against pathogens but are also able to synthesize various cytokines such as pro-inflammatory cytokines and growth factors [48]. In addition, they play an essential role in tissue homeostasis and repair [49]. In fact, two distinct subsets of macrophages exist depending on environmental factors and cell status: the classically activated macrophages M1 are polarized by components such as LPS and produce pro-inflammatory cytokines such as TNF-α, and the alternatively activated macrophages (M2) which are anti-inflammatory and immunoregulatory [50,51]. Macrophages also play a crucial role in inflammation. Thus, finding biomolecules able to counter exacerbated inflammation is of great importance, and phenolic compounds able to reduce TNF-α production are promising agents to inhibit inflammation. Here, we showed that polyamines such as spermine derivatives, when added after LPS, inhibit proinflammatory cytokine production by human monocytes and polarized macrophages into M2 cells [52,53,54]. In the same way, TDHCS and CP counteracted the LPS pro-inflammatory effect on THP-1 cells.

When tomato leaves are infested by *T. absoluta*, Kuk_A, CP and TDHCS strongly accumulate in leaves. Our experiments have shown that extracts from non-infested tomato leaves were able to diminish TNF-α production, likely because of their content in chlorogenic acid, but extracts from tomato leaves infested by *T. absoluta* failed to counteract this effect, whereas chlorogenic acid was also present. This ability of CGA to inhibit LPS-induced pro-inflammatory response was already observed in THP-1 cells and RAW 264.7 macrophages [42,55,56]. The fact that extracts from infested tomatoes leaves were less efficient than extracts from non-infested leaves suggests either that phenolamides concentration was not high enough in infested leaves to lead to an anti-inflammatory effect and/or that molecules due to *T. absoluta* infestation antagonized this effect. Further prospects should be undergone to explain this unexpected result.

Despite the fact that infested tomato leaf extract did not exhibit enhanced anti-inflammatory properties, it could be seen as an accessible source for the purification of these metabolites of interest. This is especially true for TDHCS and, to a lesser extent, for Kuk_A. These two phenolamides have been described only in potato tubers and root bark of *Lycium* sp. [57,58]. Since the concentrations of Kuk_A could reach several mg/g in *Lycium* sp. root bark, TDHCS could be found at only tens of µg/g in potato tubers, which is far less than the concentrations found in infested tomato leaves (up to one mg/g DW). Biotechnology appears also as a promising way to produce such molecules. Indeed, several bacteria strains and yeast have been engineered to express this plant-specific metabolic pathway and produce a diversity of phenolamides such as clovamide or dopamine- and serotonin-derivatives [59,60,61]. More recently, Perrin and coworkers set up a yeast strain able to produce spermidine substituted with one to three phenolic acids, giving rise to a large diversity of phenolamides some of which being new-to-nature [62]. Even if some of these structures were close to TDHCS, the biotechnological production of this specific molecule is currently blocked due to the actual gap in knowledge regarding the plant genes and enzymes catalyzing its biosynthesis. In conclusion, in addition to the identification of the anti-inflammatory property of three phenolamides, among them the rare TDHCS, our study proposes two potential ways, i.e., chemical synthesis and/or plant purification as source of pure TDHCS.

## 4. Materials and Methods

### 4.1. Plant Obtention and Growth Conditions

Tomato seeds (*S. lycopersicum* L.) of the early and dwarf cultivar “Better Bush” (VFN Hybrid, breeder: Tomato Growers) were sown and grown using a hydroponic system under controlled conditions as described in [26].

*T. absoluta* adults were provided by INRAE-ISA Sophia-Antipolis (France). They were reared on tomato plants as described in [26]. Briefly, tomato plants were grown in climatic chamber under insectarium where females laid eggs on leaves. Larvae at stage 3 (scale 1–4) were selected in order to infest young tomato plants [26].

Leaf infestation by *T. absoluta* larvae: Twenty tomato plants of 14 days old were used for the experiments and grown-cultured as previously described in [26]. Each plant was infested with 12 larvae as described in [26]. After 4 days of infestation, leaves were frozen in liquid nitrogen, and then grounded to a fine powder and stored at −20 °C.

### 4.2. Metabolite Extraction from Tomato Leaves

Metabolites were extracted from 30 mg dry powder of leaves as described in [63]. The dry powder was extracted in 1 mL 60% aqueous methanol to which 50 μL of taxifolin were added (internal standard, 2 mg·mL^−1^ methanol), then blended (1 min) and centrifuged (10 min, 2800 *g*). The extraction was repeated once, and the supernatants pooled and vacuum-dried. The residue was dissolved in 500 µL of 70% aqueous methanol, filtered (0.22 μm) and transferred to vials before U-HPLC–DAD–MS analyses.

### 4.3. Phenolamides

#### 4.3.1. General Information

CP and Kuk_A were purchased from TransMIT PlantMetaChem (Giessen, Germany). TDHCS synthesis was performed under argon atmosphere. Melting points were measured on a Kofler bench and were uncorrected. ^1^H and ^13^C NMR spectroscopic data were recorded on a Bruker Advance III 400 (400 and 100.6 MHz, respectively). Chemical shifts were reported in ppm (δ) and were measured related to the signals for the residual solvent [64]. High resolution mass spectra (HRMS) were recorded on a Bruker MicrOTOFq ESI/QqTOF apparatus. Reaction completion was checked by TLC (Thin layer chromatography) using 60 F254 (Merck) plates and were revealed with UV light (254 nm). Flash column chromatography were achieved on a Reveleris^®^ Grace equipped with 40 µm silica cartridges. The final compound was purified by semi-preparative HPLC using a 250 mm × 10 mm Thermofischer Gold column, a Gilson 321 H2 pump and a Shimadzu SPD 10A VP UV detector. Anhydrous THF and MeOH were freshly collected on a MBRAUN MB-SPS-800 solvent purification system. Dichloromethane was distilled over CaH_2_ and stocked on 4 Å molecular sieves. DMF was also stocked on 4 Å molecular sieves. Other commercial reagents were used without further purification.

#### 4.3.2. Synthesis of *N*^1^,*N*^5^,*N*^14^-tris(dihydrocaffeoyl)spermine

Methyl 1-(3,4-Dihydroxyphenyl)propionate (**2**). The titled compound was synthesized according to a slight modification of a procedure reported by Bourne et al. [39]. A solution of dihydrocaffeic acid **1** (1.000 g, 5.49 mmol) in MeOH (25 mL) was added to concentrated sulfuric acid (0.3 mL). The mixture was heated to reflux for 12 h and the solvent was evaporated to dryness. The residue was dissolved in EtOAc (20 mL) and water (10 mL) and the mixture was shaken. After separation, the organic phase was washed with 5% aqueous NaHCO_3_ solution (2 × 20 mL) and brine (2 × 20 mL). The solution was dried (MgSO_4_) and concentrated to dryness to give 1.018 g (5.19 mmol, 95% yield) of a colorless liquid which crystallized on standing. M.P. 72–74 °C. ^1^H NMR (CDCl_3_) δ 2.59 (t, *J* = 7.5 Hz, 2 H), 2.84 (t, *J* = 7.5 Hz, 2 H), 3.67 (s, 3 H), 5.30 (br s, 2 H), 6.62 (dd, *J* = 2.0, 8.0 Hz, 1 H), 6.71 (d, *J* = 2.0 Hz, 1 H), 6.77 (d, *J* = 8.0 Hz, 1 H). Other analyses were in accordance with the literature [40].

2,2-Dimethyl-1,3-benzodioxole-5-propanoic acid methyl ester (**3**). The titled compound was synthesized according to a procedure reported by Wilms et al. [40]. In a three-neck round-bottom flask equipped with a Soxhlet extractor with a reflux condenser, Methyl 1-(3,4-Dihydroxyphenyl)propionate **2** (1.000 g, 5.10 mmol) and *p*-toluenesulfonic acid monohydrate (39 mg, 0.20 mmol) were suspended in 130 mL of benzene. The Soxhlet thimble was filled with CaCl_2_. 2,2-Dimethoxypropane (0.95 mL, 7.65 mmol) was syringed in and the reaction mixture was refluxed overnight. The solvent was concentrated to a 20 mL volume and the solution was diluted with EtOAc (100 mL). The resulting solution was washed with 5% aqueous NaHCO_3_ (2 × 50 mL), water (2 × 50 mL), dried (MgSO_4_), and concentrated to dryness to give a dark orange liquid. The latter was purified by column chromatography (eluent: cyclohexane/EtOAc, 100:0 to 80:20) to give 706 mg (2.99 mmol, 59% yield) of yellow liquid. ^1^H NMR (CDCl_3_) δ 1.65 (s, 6 H, CH_3_), 2.58 (t, *J* = 8.0 Hz, 2 H, CH_2_), 2.85 (t, *J* = 8.0 Hz, 2 H, CH_2_), 3.67 (s, 3 H, OCH_3_), 6.57–6.65 (m, 3 H, H_arom_). ^13^C NMR (CDCl_3_) δ 26.0 (CH_3_), 30.9 (CH_2_), 36.2 (CH_2_), 51.7 (OCH_3_), 108.2 (CH_arom_), 108.7 (CH_arom_), 117.8, 120.6 (CH_arom_), 133.8, 146.0, 147.6, 173.5 (C=O). ESI-HRMS (pos. mode) *m*/*z*: calculated for C_13_H_17_O_4_ ([M+H]^+^): 237.1121; found : 237.1154.

2,2-Dimethyl-1,3-benzodioxole-5-propanoic acid (**4**). To a solution of the starting ester (687 mg, 2.91 mmol) in THF (10 mL) was added a solution of LiOH (209 mg, 8.72 mmol) in water (10 mL) and the mixture was stirred at room temperature for 2.5 h. The organic solvent was removed in vacuo and the remaining water was extracted with CH_2_Cl_2_ (2 × 15 mL), and then acidified to pH 4 with 5% aqueous citric acid. The resulting suspension was extracted with EtOAc (25 mL) and the organic phase was washed with water (2 × 20 mL), dried (MgSO_4_), and concentrated to dryness to give 592 mg (2.66 mmol, 92% yield) of white powder. M.P. 107–109 °C. ^1^H NMR (CDCl_3_) δ 1.66 (s, 6 H, CH_3_), 2.63 (t, *J* = 8.0 Hz, 2 H, CH_2_), 2.86 (t, *J* = 8.0 Hz, 2 H, CH_2_), 6.58–6.66 (m, 3 H, H_arom_). ^13^C NMR (CDCl_3_) δ 26.0 (CH_3_), 30.5 (CH_2_), 36.1 (CH_2_), 108.2 (CH_arom_), 108.7 (CH_arom_), 117.9, 120.6 (CH_arom_), 133.4, 146.1, 147.7, 178.8 (C=O). ESI-HRMS (neg. mode) *m*/*z*: calculated for C_12_H_13_O_4_ ([M−H]^−^): 221.0819; found: 221.0779.

*N*^1^,*N*^14^-bis(1-(4,4-dimethyl-2,6-dioxocyclohexylidene)ethyl)spermine (**5**). According to a slight modification of a procedure reported by [38], a solution of spermine (1.200 g, 5.93 mmol) in MeOH (8 mL) under argon was added a solution of DdeOH (2.344 g, 12.45 mmol) in MeOH (30 mL) and the mixture was stirred at room temperature overnight. The solution was concentrated, and the residue was directly purified by column chromatography (eluent: CH_2_Cl_2_/MeOH, 100:0 to 30:70) to give 2.200 g (4.15 mmol, 70% yield) of white wax. ^1^H NMR (CDCl_3_) δ 1.02 (s, 12 H, 2 × C(CH_3_)_2_-Dde), 1.49 (m, 6 H, NHCH_2_-C*H_2_*C*H_2_*CH_2_NH + 2 × NH), 1.82 (quint, *J* = 6.9 Hz, 4 H, 2 × NHCH_2_C*H_2_*CH_2_NH), 2.35 (s, 8 H, 4 × COCH_2_), 2.56 (s, 6 H, 2 × C=CCH_3_), 2.60 (br t, *J* = 6.3 Hz, 4 H, NHC*H_2_*CH_2_CH_2_C*H_2_*NH), 2.71 (t, *J* = 6.8 Hz, 4 H, 2 × NHC*H_2_*CH_2_CH_2_NH), 3.48 (m, 4 H, 2 × NHCH_2_CH_2_C*H_2_*NH), 13.35 (br s, 2 H, NH-Dde). Other analyses were in accordance with the literature [38].

*N*^1^,*N*^14^-bis(1-(4,4-dimethyl-2,6-dioxocyclohexylidene)ethyl),*N*^5^-*tert*-butyloxycarbonyl spermine (**6**). To a vigorously stirred solution of diprotected spermine **5** (2.131 g, 4.01 mmol) in CH_2_Cl_2_ (40 mL) was added dropwise a solution of di-*tert*-butyl dicarbonate (701 mg, 3.21 mmol) in CH_2_Cl_2_ (15 mL). The mixture was stirred under argon at room temperature overnight. The solution was concentrated, and the residue was directly purified by column chromatography (eluent: CH_2_Cl_2_/MeOH, 100:0 to 50:50) to give 1.504 g (2.38 mmol, 74% yield) of colorless oil. ^1^H NMR (CDCl_3_) δ 1.01 (s, 12 H, 2 × C(CH_3_)_2_-Dde), 1.42 (s, 9 H, *t*-Bu), 1.54 (m, 4 H, NHCH_2_-C*H_2_*C*H_2_*CH_2_NH + 2 × NH), 1.89 (m, 4 H, 2 × NHCH_2_C*H_2_*CH_2_NH), 2.34 (s, 8 H, 4 × COCH_2_), 2.54 (s, 3 H, C=CCH_3_), 2.56 (s, 3 H, C=CCH_3_), 2.68 (m, 2 H, NHC*H_2_*CH_2_CH_2_CH_2_NH), 2.78 (br t, *J* = 6.3 Hz, 2 H, NHC*H_2_*CH_2_CH_2_NH), 3.18 (br t, *J* = 6.3 Hz, 2 H, C*H_2_*NBoc), 3.29 (t, *J* = 6.8 Hz, 2 H, C*H_2_*NBoc), 3.39 (m, 2 H, NHCH_2_CH_2_C*H_2_*NH), 3.50 (m, 2 H, NHC*H_2_*CH_2_CH_2_NH), 13.40 (br s, 2 H, NH-Dde). ^13^C NMR (CDCl_3_) δ 17.9 (CH_3_), 18.0 (CH_3_), 28.4 (CH_3_ × 4), 28.6 (*t*-Bu), 30.2 (*C*H_2_CO × 4), 41.3 (CH_2_), 44.6 (CH_2_), 46.6 (CH_2_), 49.2 (CH_2_), 53.0, 80.0 (*t*-Bu C_quat_), 108.1, 155.7, 173.6 (C=O), 173.7 (C=O). ESI-HRMS (pos. mode) *m*/*z*: calculated for C_35_H_59_N_4_O_6_ ([M+H]^+^): 631.4429; found: 631.4410.

*N*^1^,*N^5^,N*^14^-tris(2,2-dimethylbenzo[d][1,3]dioxol-5-yl)propanamido,*N^10^*-*tert*-butyloxycarbonyl spermine (**8**). (a) Dde removal: To a solution of compound **6** (258 mg, 0.41 mmol) in DMF (5 mL) was added a 25% aqueous solution of hydrazine hydrate (1.00 mL) and the solution was stirred at room temperature for 20 min. The solvent was removed under vacuum and the residue was partitioned between CH_2_Cl_2_ (15 mL) and water (20 mL). The aqueous phase was extracted 5 more times with CH_2_Cl_2_ (5 mL each time) and evaporated to give 50 mg (0.16 mmol, 39% yield) of *N*^5^-*tert*-butyloxycarbonylspermine **7** as a colorless residue, which was directly involved in the next step without any further purification. (b) Coupling with dihydrocaffeic acid: to an argon-flushed solution of protected dihydrocaffeic acid **4** (129 mg, 0.58 mmol) in CH_2_Cl_2_ (8 mL) was added triethylamine (81 µL, 0.58 mmol) and the solution was cooled with an ice bath. Ethylchloroformate (55 µL, 0.58 mmol) was added, the mixture was stirred at the same temperature for 1 h, and then added to a suspension of *N*^5^-*tert*-butyloxycarbonylspermine **7** in 5 mL of CH_2_Cl_2_. The reaction mixture was stirred overnight, concentrated to dryness and dissolved in EtOAc (30 mL). The organic phase was washed with 5% aqueous citric acid (2 × 20 mL), 5% aqueous NaHCO_3_ (2 × 20 mL), water (2 × 20 mL), dried and concentrated to dryness to give 159 mg of residue, which was purified by column chromatography (eluent: cyclohexane/EtOAc, 30:70, then CH_2_Cl_2_/MeOH 98:2 to 96:4) to give 75 mg (0.08 mmol, 20% yield over two steps) of a low-melting-point white solid. ^1^H NMR (CDCl_3_) δ 1.39–1.48 (m, 13 H, *t*-Bu + NCH_2_C*H_2_*C*H_2_*CH_2_N), 1.53–1.66 (m, 22 H, acetonide CH_3_ × 6 + NCH_2_C*H_2_*CH_2_N × 2), 2.33–2.58 (m, 6 H, COC*H_2_*CH_2_Ar), 2.79–2.90 (m, 6 H, COCH_2_C*H_2_*Ar), 3.03–3.33 (m, 12 H, NC*H_2_*CH_2_C*H_2_*N × 2 + N*CH_2_* CH_2_CH_2_C*H_2_*N), 5.59 (br s, 1 H, NH), 6.52–6.65 (m, 9 H, H_arom_), 6.75 (br s, 1 H, NH). ^13^C NMR (CDCl_3_) δ 25.9 (Acetonide CH_3_), 27.5 (CH_2_), 27.8 (CH_2_), 28.5 (*t*-Bu CH_3_), 31.5 (CH_2_), 31.6 (CH_2_), 31.7 (CH_2_), 36.3 (CH_2_), 36.4 (CH_2_), 36.5 (CH_2_), 37.0 (CH_2_), 38.7 (CH_2_), 39.0 (CH_2_, 2 peaks), 42.3 (CH_2_), 45.6 (CH_2_), 46.6 (CH_2_), 47.6 (CH_2_), 80.0 (*t*-Bu C), 108.0 (CH_arom_), 108.1 (CH_arom_), 108.2 (CH_arom_), 108.7 (CH_arom_, 2 peaks), 108.8 (CH_arom_), 117.6, 117.7, 117.9, 120.5 (CH_arom_, 2 peaks), 120.6 (CH_arom_), 134.2, 134.3, 134.5, 145.8, 145.9, 146.0, 147.5, 147.6, 147.7, 156.5 (carbamate C=O), 172.3 (amide C=O), 172.4 (amide C=O), 172.9 (amide C=O). ESI-HRMS (pos. mode) *m*/*z*: calculated for C_51_H_71_N_4_O_11_ ([M+H]^+^): 915.5114; found: 915.4943.

*N*^1^,*N*^5^,*N*^14^-tris(dihydrocaffeoyl)spermine (**9**). To a solution of compound **8** (75 mg, 0.08 mmol) in CH_3_CN (10 mL) was added 1 M aqueous HCl solution (10 mL) and the mixture was heated at 60 °C for 48 h. The solvent was evaporated, and the residue was purified by reversed-phase HPLC (eluent: H_2_O, 0.02% trifluoroacetic acid/MeOH, 0.02% trifluoroacetic acid 100:0 to 40:60 in 50 min) to give 24 mg (0.03 mmol, 37% yield) of a white low-melting-point solid as a trifluoroacetate salt. ^1^H NMR (MeOD) δ 1.39–1.65 (m, 6 H, NCH_2_C*H_2_*C*H_2_*CH_2_N + NCH_2_C*H_2_*CH_2_N), 170–1.80 (m, 2 H, NCH_2_C*H_2_*CH_2_N), 2.35–2.91 (m, 16 H, COC*H_2_*C*H_2_*Ar × 3 + C*H_2_*NHC*H_2_*), 3.04–3.33 (m, 8 H, C*H_2_*NHCO × 4), 6.48–6.58 (m, 3 H, H_arom_), 6.61–6.72 (m, 6 H, H_arom_). ^13^C NMR (CDCl_3_) δ 24.6 (CH_2_), 24.7 (CH_2_), 25.7 (CH_2_), 27.0 (CH_2_), 27.7 (CH_2_), 28.5 (CH_2_), 29.9 (CH_2_), 31.9 (CH_2_), 32.2 (CH_2_), 32.3 (CH_2_), 32.4 (CH_2_), 37.0 (CH_2_), 36.2 (CH_2_), 36.4 (CH_2_), 36.5, (CH_2_), 37.7 (CH_2_), 37.9 (CH_2_), 38.3 (CH_2_), 38.4 (CH_2_), 39.2 (CH_2_), 39.3 (CH_2_), 45.0 (CH_2_), 45.9 (CH_2_), 46.4 (CH_2_), 47.1 (CH_2_), 116.3 (CH_arom_), 116.4 (CH_arom_), 116.5 (CH_arom_), 116.6 (CH_arom_), 116.7 (CH_arom_), 116.8 (CH_arom_, 3 peaks), 120.6 (CH_arom_), 120.7 (CH_arom_), 120.8 (CH_arom_), 120.9 (CH_arom_), 133.4, 133.6, 133.7, 133.8, 134.0, 144.5, 144.6, 144.7, 146.1, 146.2, 146.3, 175.1, 175.4, 175.6, 176.6, 176.8. ESI-HRMS (pos. mode) *m*/*z*: calculated for C_37_H_51_N_4_O_9_ ([M+H]^+^): 695.3651; found: 695.3732.

### 4.4. Analysis of Phenolamides Content by UPLC Analysis

Tomato leaf extracts were analyzed using a U-HPLC system (Prominence, Shimadzu, Kyoto, Japan) equipped with a binary solvent delivery pump connected to a diode array detector (SPDM20A, Shimadzu, Kyoto, Japan) and a single quadrupole mass spectrometer (LCMS2020, Shimadzu, Kyoto, Japan). An aliquot of 3 µL extract was separated on a C18 Zorbax Eclipse Plus (150 × 2.1 mm, 1.8 µm) column (Agilent, Palo Alto, CA, USA) using a gradient elution from 1 to 50% MeOH 0.1% formic acid (FA) in 7.1 min, then 99% MeOH 0.1% FA in 0.8 min, with a flow rate of 430 µL/min. The column was rinsed for 2 min with 99% MeOH 0.1% FA and re-equilibrated to the initial conditions for 2 min prior to the next run. The main metabolites were identified using the mass and retention time information and according to [26,37] and compared to available commercial standards. Quantification was based on the area under the peak determined at 280, 320 and 350 nm and expressed relative to calibration curves (caffeic acid for caffeoylhexaric acid; ferulic acid for feruloylquinic acid, chlorogenic acid, rutin for rutin, apiofuranosylrutin and kaempferol rutinoside, kukoamine A for TDHCS and kukoamine A; Figure S4). *t*-Tests were used to identify statistically significant differences in the metabolite concentrations between healthy and *T. absoluta*-infested tomato organs (*p* ≤ 0.05).

### 4.5. Antibacterial Activity

#### 4.5.1. Bacteria Strains and Culture Conditions

The bacterial strains used in this study included Gram-positive bacteria: *Enterococcus faecalis* (ATCC 29212), *Staphylococcus aureus* (ATCC 29213), as well as Gram-negative ones: *Escherichia coli* (ATCC 25922), *Klebsiella pneumoniae* (ABC 42), *Enterobacter cloacae* (ABC 291), *Pseudomonas aeruginosa* (ATCC 27853) and *Acinetobacter baumannii* (ABC 14). All strains were kindly provided by the ABC Platform^®^ Bugs Bank. All bacterial strains were cultured on Mueller Hinton Agar (MHA, Difco 225250), and the antibacterial tests were realized on Mueller Hinton Broth (MHB, Difco, 275730).

#### 4.5.2. MIC Determination

Antibacterial activity was evaluated by determining minimum inhibitory concentration (MIC) as previously described [65]. MICs were determined by broth microdilution method recommended by the CLSI (NCCLS, 2003). For MIC determination, suspensions were prepared by suspending one isolated colony from Mueller Hinton plates in 5 mL of Mueller Hinton Broth. After 24 h of growth, the suspensions were diluted in distilled water to obtain a final inoculum of 5 × 10^5^ to 5 × 10^6^ CFU/mL. Twofold serial dilutions of drugs were prepared in Mueller Hinton Broth in 96-well plates (Greiner, 650161), starting from a stock solution of 4096 mg/L. An equal volume of bacterial inoculum was added to each well on the microtiter plate containing 0.05 mL of the serial compound dilutions. After incubation for 18–24 h at 35 °C, the MICs were determined with an ELISA reader (read at 540 nm, Multiskan EX, Thermo Electron Corporation, France) as the lowest concentration of compound whose absorbance was comparable with the negative control wells (broth only or broth with drug, without inoculum). Results were expressed by means of three independent determinations.

### 4.6. Anticancer Activity

The human MCF-7 breast cancer cell line (ATCC HTB-22), and the human colonic Caco-2 cell line (ATCC HTB-37) were used for the anticancer assays.

#### 4.6.1. Cell Culture and Treatments

Both cell lines were cultured in Eagle’s minimal essential medium supplemented with 1% non-essential amino acids, 1% L-glutamine and 10% fetal bovine serum at 37 °C in a 5% CO_2_ humidified atmosphere. All products were purchased from SIGMA-Aldrich (Saint-Quentin-Fallavier, France).

#### 4.6.2. Cell Proliferation Assay

Anticancer activity was evaluated as the capability of tested compounds to inhibit the cell growth of cancerous cells (i.e., MCF-7 and Caco-2 cells). Assays were carried out using a commercially available cell proliferation reagent [3-(*4,5-dimethylthiazolyl-2-yl*)-2,5-diphenyltetrazolium bromide] (MTT) as described previously [66,67]. The assay is based on the cleavage of the tetrazolium salt MTT by active mitochondrial dehydrogenases to produce an insoluble purple formazan salt. Since this conversion only occurs with viable cells, it directly correlates with cell count. Cells were plated at 10^4^ cells per well in 96-well tissue culture plates (83.1835, Sarstedt) and grown for 48 h at 37 °C in a 5% CO_2_ atmosphere. Then, medium was discarded and replaced by fresh medium containing increasing amounts of compounds previously dissolved in dimethylsulfoxide (DMSO). The final concentration of DMSO never exceeded 2% of the final volume. After 24 h incubation, the medium was discarded and cells were washed with PBS. 100 μL of medium containing 0.5 mg/mL MTT prepared previously in PBS were added to each well and the plates were incubated in a humidified atmosphere with 5% CO_2_ at 37 °C for 4 h. Then, formazan crystals were dissolved by the addition of 100 μL of SDS (100 μg/mL SDS in PBS, with 445 µL HCl 0.01 M), followed by incubation for 4 h at 37 °C. Finally, the absorbance was measured at 540 nm vs. 690 nm using a 96-well plate reader (Multiskan EX, Thermo Electron Corporation, Villebon sur Yvette, France). Experiments were repeated three times.

### 4.7. Anti-Inflammatory Activity

The THP-1 human monocytic leukemia cell line was purchased from the European Collection of Authenticated Cell Cultures (ECACC). THP-1 cells were used for cytotoxic and anti-inflammatory assays.

#### 4.7.1. Cell Culture and Treatments of THP-1 Cells

THP-1 cells were cultured in RPMI 1640 medium supplemented with 10% heat inactivated fetal calf serum, 100 U/mL penicillin, 100 µg/mL streptomycin, 10 mM HEPES, 2 mM L-glutamine, 1 mM sodium pyruvate, and 1 × non-essential amino-acids at 37 °C under 5% CO_2_. All products were purchased from SIGMA-Aldrich (Saint-Quentin-Fallavier, France). THP-1 cells, at a density of 0.8 × 10^6^ cells/mL in 24-wells plates, were differentiated for 3 days into macrophages by adding in the cell culture medium 20 nM of phorbol myristate acetate (SIGMA-Aldrich). Then, differentiated THP-1 cells were incubated for 24 h with 100 ng/mL of LPS added one hour before or after a pure polyphenol or tomato leaf extract. CP, Kuk_A and TDHCS were dissolved in RPMI 1640 medium and sterilized with 0.2 µM syringe filters (SIGMA-Aldrich). Thus, CP, Kuk_A and TDHCS were tested at 15, 30, 45 and 60 µM. Tomato leaf extracts were used at 50, 100 and 150 µg/mL representing, respectively, a content in chlorogenic acid of 15, 30 and 45 µg/mL. Chlorogenic acid was used as standard at 15, 30 and 45 µg/mL (SIGMA-Aldrich).

#### 4.7.2. THP-1 Cell Viability Measurement

After 24 h of incubation with LPS and/or tomato leaf extracts or pure phenolic compounds (CGA, CP, Kuk_A or TDHCS), differentiated THP-1 cell viability was determined using the crystal violet assay. THP-1 cells were washed twice with phosphate buffer saline (PBS), incubated with 0.1% crystal violet for 20 min at ambient temperature and then, carefully washed twice with PBS. Finally, cells were lyzed with 10% acetic acid for 20 min at ambient temperature. Cell contents were homogenized and analyzed by spectrophotometry at 595 nm with a multilabel counter (Wallac-1420, Perkin Elmer, Boston, MA, USA). Each experiment was performed in triplicates.

#### 4.7.3. TNF-α Quantification from THP-1 Cell Culture Supernatants

After 24 h of treatment with LPS and/or tomato leaf extracts or pure phenolic compounds, cell culture supernatants were harvested in sterile conditions, centrifuged to remove dead cells, and stored at −80 °C until ELISA tests [41]. TNF-α concentrations were determined using the Human TNF-alpha Quantikine ELISA Kit (R&D Systems, BioTechne Brands, Rennes, France). Assays were performed according to the instructions of the manufacturer, in duplicate, and repeated three to four times. Plates were read at 450 nm with a multilabel counter (Wallac-1420, Perkin Elmer, Boston, MA, USA).

### 4.8. Data Analysis

For the study of phenolic composition in tomato leaf extracts, the values were the means of six replicates. For the comparison of metabolite concentrations between healthy and *T. absoluta*-infested tomato organs, *t*-tests were used to identify statistically significant differences (*p* ≤ 0.05) using R studio. For THP-1 cell viability and anti-inflammation studies, six independent experiments were performed for each phenolamide as well as for each tomato leaf extract. T-tests were again used to identify statistically significant differences (*p* ≤ 0.05) using GraphPad Prism 9.

## 5. Conclusions

In this study, we propose a new synthesis of the phenolamide *N*^1^*,N*^5^*,N*^14^-tris(dihydrocaffeoyl)spermine (TDHCS) from its spermine motif. To our knowledge, it is the second time that its synthesis is described. We demonstrate that the three phenolamides, Kuk_A, CP and TDHCS exert a moderate antimicrobial activity against different bacterial strains. We also establish that these three phenolamides are able to decrease pro-inflammatory response initiated by LPS in THP-1 cells differentiated into macrophages. Furthermore, added one hour before LPS, they render THP-1 cells less sensible to the LPS pro-inflammatory action. Of the three phenolamides, CP and TDHCS were the most powerful inhibitors of LPS action. Taken together, these data show that these phenolamides could be interesting alternatives to conventional drugs.

## Data Availability

Phenolic samples are available in a delay of 6 month. Others samples are of short-term conservation and cannot be stored. Data are available on demand to the authors.

## Supplementary Materials

The following supporting information can be downloaded at: https://www.mdpi.com/article/10.3390/molecules28041552/s1, Figure S1. Minimum inhibitory concentrations (MICs) in mg/L of kukoamine A (Kuk_A), caffeoylputrescine (CP), and *N*^1^*,N*^5^*,N*^14^-tris(dihydrocaffeoyl)spermine (TDHCS) against listed bacterial strains. Figure S2. TDHCS cytotoxic activity on MCF-7 cells. Mean of eight points were calculated and standard deviations are represented. Figure S3. TDHCS cytotoxic activity on Caco-2 cells. Mean of eight points were calculated and standard deviations are represented. Figure S4. Calibration curves for metabolites quantification at specified wavelength.
