# Supplementary material for: Characterization of Biological Properties of Individual Phenolamides and Phenolamide-Enriched Leaf Tomato Extracts
Source: Molecules. 2023 Feb 6;28(4):1552. doi: 10.3390/molecules28041552 (PMC9966281; doi:10.3390/molecules28041552)
Supplement: Supplementary file 1 [file molecules-28-01552-s001.zip › molecules-2164619-supplementary.pdf]

Supplementary materials

Characterization of biological properties of individual phenolamides and phenolamide-enriched leaf tomato extracts

Marwa Roumani [1,†], Armelle Ropars [2,†], Christophe Robin [1], Raphaël E. Duval [3], Jean-Pol Frippiat [2], Michel Boisbrun [3,*] and Romain Larbat [1,4,*]

[1] Université de Lorraine, INRAE, LAE, F-54000 Nancy, France
[2] Université de Lorraine, SIMPA, F-54000 Nancy, France
[3] Université de Lorraine, CNRS, L2CM, F-54000 Nancy, France
[4] Institut Agro, University of Angers, INRAE, IRHS, SFR QUASAV, F-49000 Angers, France
* Correspondence: michel.boisbrun@univ-lorraine.fr (M.B.); romain.larbat@inrae.fr (R.L.)
† These authors contributed equally to this work.

Table of contents

Figure S1. Minimum inhibitory concentrations (MICs) in mg/L of Kukoamine A (Kuk_A), caffeoylputrescine (CP), and *N^1,N^5,N^{14}*-tris(dihydrocaffeoyl)spermine (TDHCS) against listed bacterial strains.

Figure S2. TDHCS cytotoxic activity on MCF-7 cells. Mean of eight points were calculated and standard deviation are represented.

Figure S3. TDHCS cytotoxic activity on Caco-2 cells. Mean of eight points were calculated and standard deviation are represented.

Figure S4. Calibration curves for metabolites quantification at specified wavelength

Bacterial strains	Kuk_A (mg/L)	CP (mg/L)	TDHCS (mg/L)
Staphylococcus aureus	**256**	**512**	>1024
Enterococcus faecalis	1024	1024	>1024
Pseudomonas aeruginosa	1024	>1024	>1024
Escherichia coli	1024	>1024	>1024
Klebsiella pneumoniae	>1024	>1024	>1024
Acinetobacter baumannii	512	>1024	**256**
Enterobacter cloacae	1024	>1024	>1024

Figure S1. Minimum inhibitory concentrations (MICs) in mg/L of Kukoamine A (Kuk_A), caffeoylputrescine (CP), and N^1,N^5,N^{14}-tris(dihydrocaffeoyl)spermine (TDHCS) against listed bacterial strains.

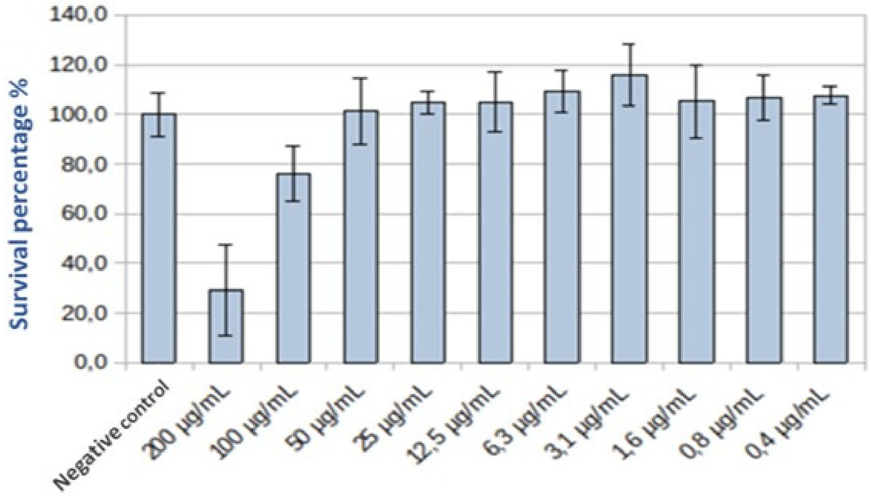

Figure S2. TDHCS cytotoxic activity on MCF-7 cells. Mean of eight points were calculated and standard deviation are represented.

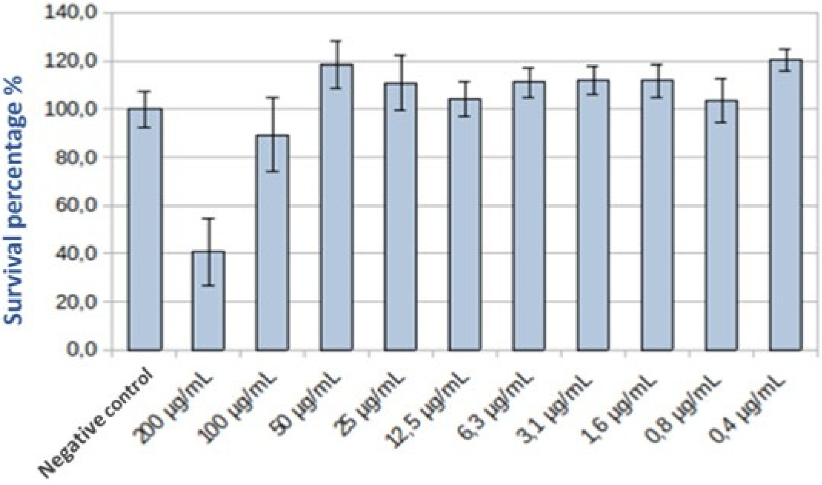

Figure S3. TDHCS cytotoxic activity on Caco-2 cells. Mean of eight points were calculated and standard deviation are represented.

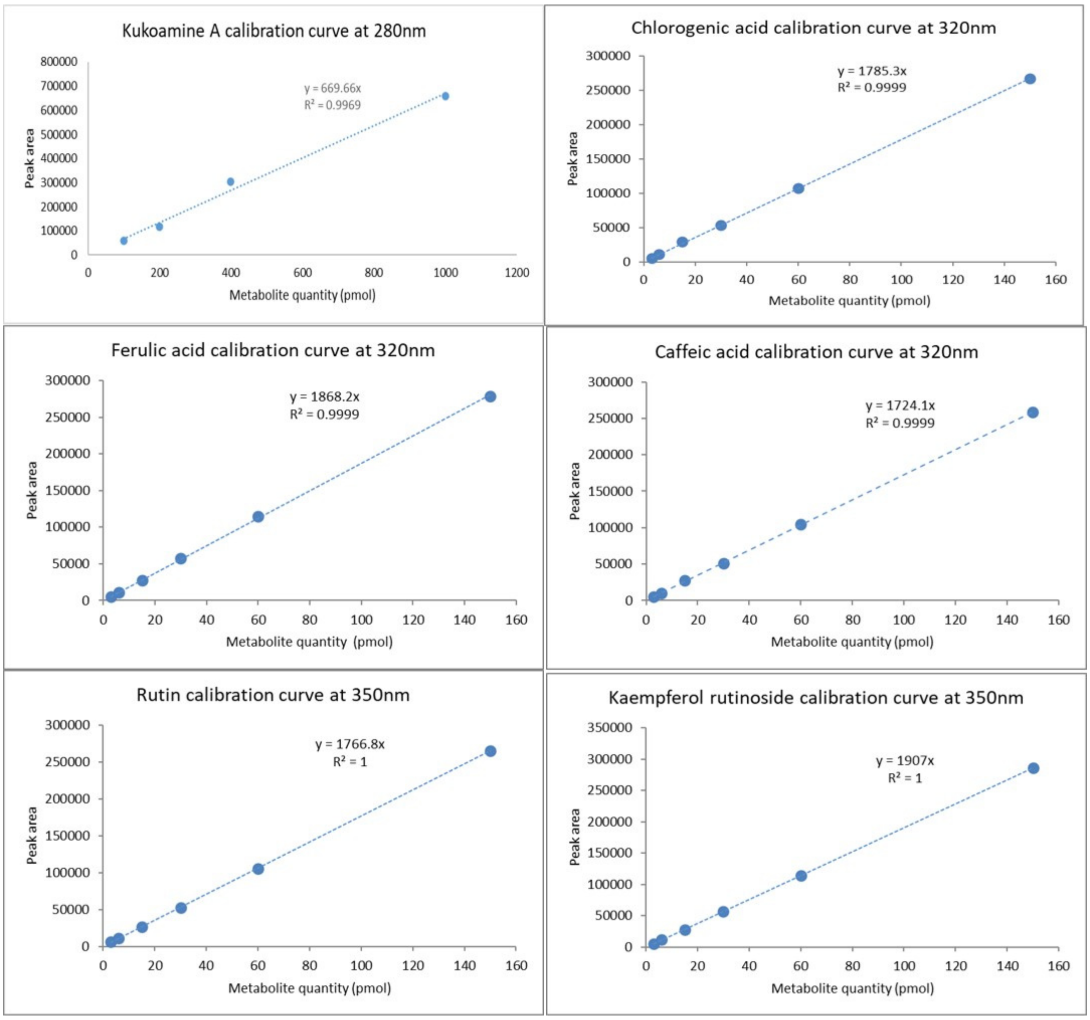

Figure S4. Calibration curves for metabolites quantification at specified wavelength
